# Cross domain fusion in power electronics dominated distribution grids

**DOI:** 10.1007/s00287-022-01495-8

**Published:** 2022-10-06

**Authors:** Pugliese Sante, Olaf Landsiedel, Johannes Kuprat, Marco Liserre

**Affiliations:** 1 Chair of Power Electronics, Kiel University, Kiel, Germany; 2 Distributed Systems, Department of Computer Science, Kiel University, Kiel, Germany

## Abstract

In the near future, a drastic change in the structure of the electric grid is expected due to the increasing penetration of power electronics interfaced renewable energy sources (e.g. solar and wind), highly variable loads (e.g. electric vehicles and air conditioning) and unexpected energy demanding events (e.g. pandemics or natural disasters). Energy balancing management, voltage and frequency stability, reduced system inertia, grid resilience to fault conditions, and power quality of the supply are a few of the main challenges in the future power electronics dominated grids. Power electronics can solve these by integrating information and communication technology in new intelligent, highly reliable, and efficient devices like smart transformers. Smart transformers can increase the power flow flexibility by enabling the correct meshed-hybrid grid operations, as long as load mission and power generation profiles are known. Those profile are generally driven by heterogeneous, highly sparse and often incomplete data that belong to different domains. This article highlights the necessity of new approaches and models to identify patterns and events of interest that can serve as a common base. The resulting patterns can then be cross-fused in a common language and form the basis of further data analytics in future distribution grids.

## Introduction

Global warming and the necessity of CO_2_ emission reduction are moving the energy generation from fossil-based to renewable-based solutions like photovoltaic (PV) and wind-turbine (WT) resources. A drastic change in the structure of the actual electrical grid, which will experience an increasing penetration of power electronics (PE) interfaced renewable energy sources (RES) and loads (e.g. electric vehicles), is expected in the near future.

This opens up several challenges either at power system level in terms of control for grid stability (e.g. frequency and voltage control) due to the volatile nature of PV and WT, or at power converter level in terms of efficiency and reliability of the power electronics interface. Power converters are in fact prone to physical failure in their sensitive components like power semiconductor and capacitors, so the manufacturer must ensure a high level of reliability on such components and monitoring of the state of health of the system to prevent possible failure.

The reliability of power electronic converters is of the utmost importance due to the progressing conversion towards a power electronics dominated grid. For a highly reliable operation, knowledge of the remaining useful lifetime and including prognostic maintenance of the converter is essential. One of the most vulnerable components of a converter is the power module. However, state-of-the-art analytical lifetime models to derive the health status of power modules are solely based on results of accelerated power cycling tests [1]. These characterize the maximum number of cycles power modules can withstand at a certain thermal loading (mean and swing of the junction temperature). Even though considering these quantities is reasonable, given that they describe the root cause of the power module degradation, it has been shown that lifetime models based on accelerated power cycling test results are inaccurate [2]. This is also because the lifetime of power modules depends on many more circumstances, e.g. the duration of the thermal cycles, to name just one of them [3]. Characterizing lifetime models under all necessary circumstances to achieve a precise estimation would require tremendous time and financial expenditure. Taking into account the fact that product life cycles are becoming shorter enhances the need for alternative approaches to derive lifetime models for power modules.

**Fig. 1:**
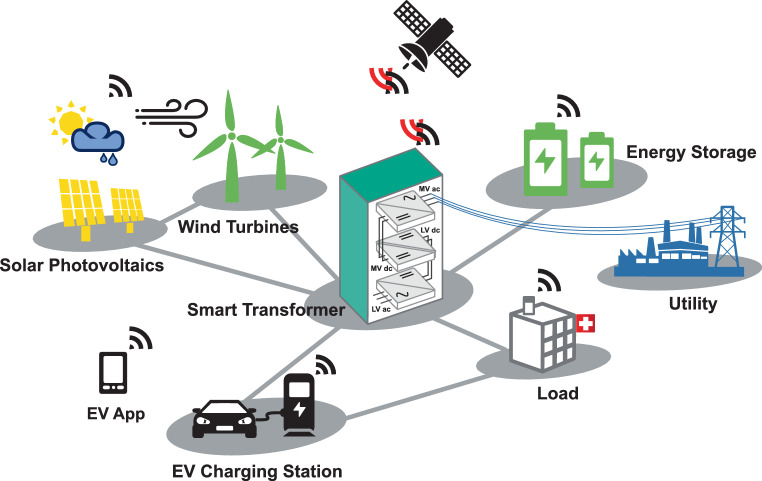
Smart Transformer operation and control based on weather forecasting data, electrical power load profile estimation in meshed and hybrid grids

At power system level, the high penetration of renewable energies and electric vehicle (EV) charging stations will add new constraints in terms of voltage limit violation and current congestion on the low-voltage network, resulting in a reduced resilience of the grid to react to a fault condition and in a reduced power quality of the supply. To avoid or postpone the reinforcements of the traditional assets in distribution grids (e.g. a higher power rating of the distribution transformer and larger cables), the network of the future should integrate intelligent devices like the smart transformers (STs) proposed in [4]. The ST is a medium-voltage (MV) to low-voltage (LV) power electronics-based transformer with smart control functionality that can address all the aforementioned challenges within a single solution. In radial networks, it has already been demonstrated that the ST is capable of providing services to electric grids, e.g. voltage regulation on MV and LV sides independently, to avoid voltage violations, active and reactive power flow control, power quality improvement. Moreover, the ST can provide direct current (dc) connectivity, both at MV and LV side, for dc grids and energy storage systems (ESSs), as shown in Fig. 1. The use of dc distribution reduces the number of power-conversion stages and the distribution losses, while increasing the power flow flexibility. In this context, the ST is the enabler of hybrid grids controlling the power flow between the alternating-current (ac) and dc grids and providing advanced services to both of them.

In fact, apart from radial operation, the ST can flexibly operate in various meshed-grid configurations: meshed at the LVac bus bar or as a ring grid at the end of the LVac feeder, meshed in a hybrid way at the LVac line together with the LVdc line, and distinct power grid regions meshed through the MVdc line [5]. The internal ST control structure depends on the grid configuration, e.g. radial or meshed, thus, when the grid configuration is changed or a grid fault occurs, the corresponding control strategy needs to be updated to ensure proper grid operations.

As an example, the voltage-variation minimization control in a LVac ring distribution grid, also known as a loop one [5], is possible through the ST active and reactive power injection. The control aims to minimize the total voltage variation by identifying the bus voltage sensitivity according to [6] and by selecting the correct active and reactive set-points. In real grids, the voltage profile along the feeder is also affected by the location of the loads and distributed generators within the network. Thus, the selection of the ST active and reactive power set-points needs to consider the location as well as the power profiles of the installed load and distributed generators to guarantee voltage limit compliance in all the network nodes, and thus to ensure grid voltage stability.

The existing research gaps and challenges in ST control management are the reference set point definition, the design of the central controller and communication system in the meshed grid configuration to optimize grid performance in terms of line loss reduction, voltage limit compliance and overload reduction.

## Approaches connected to the main idea of cross domain fusion in power converters

**Fig. 2:**
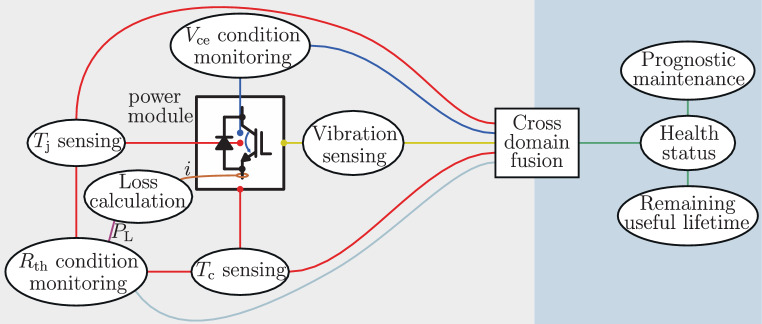
Health status derivation of power modules with cross domain fusion of stressors and condition monitoring parameters

For a highly reliable operation, knowledge of the remaining useful lifetime and including prognostic maintenance of the converters is essential. A promising opportunity would be to move from a lifetime estimation model in an open loop manner to a closed loop lifetime observer approach (Fig. 2). In this lifetime observer approach condition monitoring parameters, such as the collector emitter on-state voltage and the thermal resistance, could be used to correct the estimation by the lifetime model, which gets the stressors, such as temperatures and vibration, as feed forward. This lifetime observer approach would be a realization of cross domain fusion, which would provide an accurate observation of the health status of power modules. Accurate knowledge of the health status and its progression would then enable determining of the remaining useful lifetime and applying prognostic maintenance to achieve a more reliable electric grid of the future.

The proposed lifetime observer would rely on a hybrid model, which combines the analytical lifetime model derived based on results of accelerated power cycling tests with a data-driven model for the correction term via the condition monitoring parameters. The data-driven model, which would obtain the data from operating systems in field, would be needed to adjust the evolution of the condition monitoring parameters in dependency on the health status of the power modules for a specific application and would optimally start from a previous scientific characterization of this dependency.

## Approaches connected to the main idea of cross domain fusion in ST based meshed grids

The ST optimization algorithm leads to the meshed configuration, which minimizes a specific cost function (overload, power losses, voltage limitation); however, it depends on the knowledge of load mission and power generation profiles. Typically, these profiles are representations of electrical quantities over time whose variation is related to non-electrical phenomena, as shown in Fig. 1 (e.g. weather forecasting data, hospitalization projections in the healthcare sector, electric vehicle charging demand, etc.).

### Cross domain fusion between weather forecast data and power production

Photovoltaic (PV) power production is mostly influenced by solar irradiance and operating temperature, which in turn depends on the environment temperature. This prompted the idea to use smart metering solutions based on available internet weather forecast to predict with high accuracy the power production of PV panels in an ST supplied LV distribution grid. This would be possible without the need for extra local power sensors and communication systems between PV and ST. The role of information and communication technology (ICT) is the definition of models from data that can be used by the ST to predict power production. This prediction would be used for a correct online rescheduling of the hybrid grid configuration.

### Cross domain fusion between COVID-19 pandemic data and power loads

Electrical power load profiles are typically related to the specific distribution grid under examination; however, their time and spatial distributions are becoming ever more influenced by social aspects, and in recent years the COVID-19 pandemic has exacerbated this trend.

An example is given by the healthcare and hospital sectors. On a global scale, energy demands have decreased during the COVID-19 pandemic, but for the healthcare sector energy demands may be higher than usual due to the necessity of improved cleaning air-conditioning systems, increased numbers of intensive care beds and increased numbers of hospitalizations. In [7] a modeling of hospital energy is proposed to assess the economic costs of COVID-19 infection control interventions. The hospital energy model focuses on two components for evaluating the COVID-19 energy impact: projections for the number of COVID-19 patients over time, and the analysis of the energy usage of each intervention. The projections for COVID-19 patients were determined using an agent-based epidemiological model (ABM) developed to inform the state of Maryland about bed utilization in the Johns Hopkins Medical Institutions healthcare-system [8]. The ABM uses dynamics of compartmental models to capture infectious disease spread within a population. The hospitalization projections allowed one to predict the demand for beds and ventilators and to provide the energy/cost analysis for pandemic responses in acute care hospitals. From this example it is clear that smart power converters provided by ICT solutions can be successfully used for energy balance management, opening the path towards modern and highly PE dominated distribution grids. This includes power system controls supported by fifth-generation (5G) wireless networks enabling quick two-way response and feedback between each device in the grid [9], as shown in Fig. 1.

### Cross domain fusion in grid distribution dominated by EV charging stations

The political and societal assets that are moving towards more CO_2_ friendly mobility and the smart management of the EV charging station in the LV distribution grid is a domain of cross fusion. From a power management point of view, the ICT plays an important role providing access to crucial information for the prediction of load profile over time evolution in EV charging parks. Examples of data exchanged between EV charging parks and the ST are the state of charge (SOC) of the vehicle battery at the beginning of the charging process, the final SOC requested by the customer and the charging slot time reserved online through mobile apps. In this scenario it would be possible to precisely estimate the load profile hours in advance, and furthermore, in the case of vehicle-to-grid (V2G) services, the ST can rely also on vehicle batteries for a more flexible power management.

## Research challenges from a computer science perspective

In the context of Cross-Domain Fusion in the domain of smart power electronics and distribution grids, we see four challenges from a Computer Science perspective: (1) Heterogeneous data, (2) large volumes of data on constrained devices, (3) distributed data sources, and (4) privacy sensitive data.

### Heterogeneous data

Intelligent transformers in houses and grid control stations collect multivariate time-series data including, for example, voltage, phase and load. Further, secondary data such as temperature and system health are collected. Commonly, operating as continuous, long-term deployments, sensors provide data at high resolution in time and for a long duration. However, each sensor can only cover a limited area, such as, for example, a single transformer or one component of it. In contrast, other data sources, such as weather forecasts, lack this fine-grained granularity and instead commonly cover large geographic regions. Moreover, any forecast, be it a weather forecast or predictions of EV usage, inherently includes uncertainties. Finally, data concerning unexpected events such as pandemics or (natural) disasters, is often incomplete, inaccurate, highly sparse, and untrusted. Due to their different modalities, time-series from intelligent transformers and completing data from forecasts and unexpected events cannot be directly fused. Instead, each need to be analyzed to identify patterns and events of interest that can serve as a common base for fusion. The resulting patterns can then be fused and form the basis of further data analytics and predictions.

### Large volumes of data on constrained devices

An intelligent power grid in a city collects billions of data points per second, while each device is often connected via a constrained communication uplink, such as, for example, LoRa (Cycleo, Grenoble, France), a cellular technology, or via satellite. Especially in rural areas, cellular technologies provide low-data rate links if even available. For these reasons, for example, wind turbines in Europe are by default equipped with satellite links and not cellular links [10].

Overall, data is coming into the system or is generated by the system at a much higher velocity than the outgoing communication links can handle. This mismatch acts as a further motivator for cross-domain fusion: due to constrained links, data from nearby sensors has to be aggregated into high-level representations such as patterns to compactly represent events of interest. Such compact representations are significantly more resource-efficient in the context of communication than the raw data [12].

### Distributed data

**Fig. 3:**
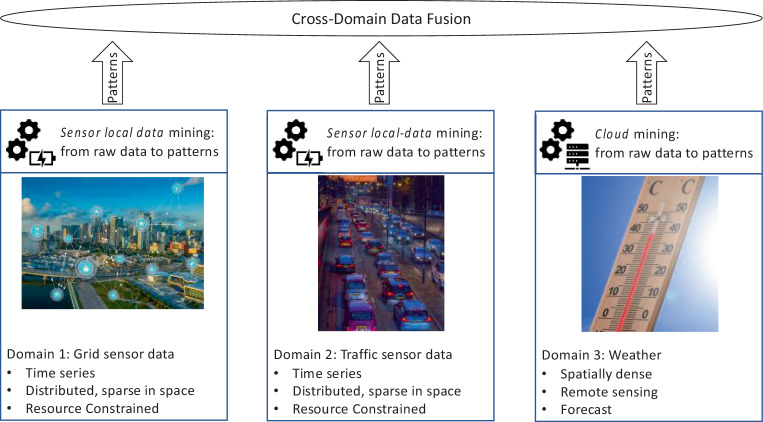
Cross-Domain Data Fusion from three domains: Power grid, road traffic, and weather. Image source: piqsels.com, image license: CC0, “No Rights Reserved”

The overall application setting is a distributed one with a built-in hierarchy. Distributed smart transformers form a distributed system with strong resource-constraints in terms of compute-power, communication capabilities, and energy. In contrast, the forecasts such as weather data or predictions of EV usage are commonly available at a location with vast compute and communication capabilities. Due to limited communication links, data has to be aggregated into compact higher-level representations, i.e., patterns. A pattern is a higher-level representation of an event of interest, such as, for example, a transformer detecting an imbalance in the grid or a misbehaving component [11]. Patterns can be identified from data of one or more sensors in close proximity. Moreover, communicating compact patterns instead of raw data limits the load on constrained communication systems [14] (see Fig. 3).

### Privacy sensitive data

Data in the above application settings are highly privacy-sensitive. For example, data on the electrical load patterns of a household gives detailed insights into the behavior of the individual members [13], such as daily routines including sleeping patterns and times of absence, i.e., travel. Similarly, the sensor data of a smart transformer give detailed insights into its operational patterns, which its vendor commonly prefers not to reveal. Finally, the power grid is a critical infrastructure and insights into the operational processes of the grid operator should not be revealed in the data. We argue that patterns, i.e., the aggregation of raw data into patterns of interest, can serve as abstraction-level for data handling and fusion. Such aggregated data are—if aggregated correctly—significantly less privacy sensitive than the raw data, representing a further reason to aggregate data as close to the source as possible and not at the point of fusion. Cross-domain data fusion is a necessity in intelligent power grids, as (1) data is heterogeneous, (2) too large to be transported, (3) widely distributed, and (4) privacy sensitive. We argue that while each property alone demands cross-domain fusion, their combination makes it a necessity.

## Conclusion

Power electronics is a key enabling and indispensable technology for an efficient, robust and resilient electrification of future energy systems, which will be strongly based on fluctuating renewable energy production mainly from wind and sun. Smart power converters equipped with ICTs can significantly improve energy management based on optimization algorithms (overload, power losses, voltage limitation, etc.), which includes holistic models extracted from cross-domain fused data. Overall, we argue that this article underlines the need for new methodologies for resource-efficient and distributed cross-domain data fusion in the context of power electronics. These will (1) serve as a common base in time and space for the fusion of heterogeneous data and (2) ensure that patterns are sufficiently small in size to be transmitted efficiently in resource-constrained settings.
